# Global COVID-19 Vaccine Acceptance: A Systematic Review of Associated Social and Behavioral Factors

**DOI:** 10.3390/vaccines10010110

**Published:** 2022-01-12

**Authors:** Choudhary Sobhan Shakeel, Amenah Abdul Mujeeb, Muhammad Shaheer Mirza, Beenish Chaudhry, Saad Jawaid Khan

**Affiliations:** 1 Department of Biomedical Engineering, Faculty of Engineering, Science, Technology and Management, Ziauddin University, Karachi 74600, Pakistan; amenah10357@zu.edu.pk (A.A.M.); shaheer.mirza@zu.edu.pk (M.S.M.); sj.khan@zu.edu.pk (S.J.K.); 2 School of Computing and Informatics, University of Louisiana at Lafayette, Lafayette, LA 70504, USA; beenish.chaudhry@louisiana.edu

**Keywords:** COVID-19, vaccine acceptance, vaccine hesitancy, associated factors, systematic review, global variations

## Abstract

COVID-19 vaccines have met varying levels of acceptance and hesitancy in different parts of the world, which has implications for eliminating the COVID-19 pandemic. The aim of this systematic review is to examine how and why the rates of COVID-19 vaccine acceptance and hesitancy differ across countries and continents. PubMed, Web of Science, IEEE Xplore and Science Direct were searched between 1 January 2020 and 31 July 2021 using keywords such as “COVID-19 vaccine acceptance”. 81 peer-reviewed publications were found to be eligible for review. The analysis shows that there are global variations in vaccine acceptance among different populations. The vaccine-acceptance rates were the highest amongst adults in Ecuador (97%), Malaysia (94.3%) and Indonesia (93.3%) and the lowest amongst adults in Lebanon (21.0%). The general healthcare workers (HCWs) in China (86.20%) and nurses in Italy (91.50%) had the highest acceptance rates, whereas HCWs in the Democratic Republic of Congo had the lowest acceptance (27.70%). A nonparametric one-way ANOVA showed that the differences in vaccine-acceptance rates were statistically significant (H (49) = 75.302, *p* = 0.009*) between the analyzed countries. However, the reasons behind vaccine hesitancy and acceptance were similar across the board. Low vaccine acceptance was associated with low levels of education and awareness, and inefficient government efforts and initiatives. Furthermore, poor influenza-vaccination history, as well as conspiracy theories relating to infertility and misinformation about the COVID-19 vaccine on social media also resulted in vaccine hesitancy. Strategies to address these concerns may increase global COVID-19 vaccine acceptance and accelerate our efforts to eliminate this pandemic.

## 1. Introduction

The COVID-19 pandemic has impacted many aspects of our everyday lives and changed the socio-economic fabric of the entire world [1,2,3,4]. The COVID-19 disease is caused by the highly contagious severe acute respiratory syndrome coronavirus 2 (SARS-CoV-2) and, at the time of its outbreak, no vaccine was available to prevent individuals from contracting the infection. Therefore, countries had to take stringent measures in order to contain the infection, including nation-wide lockdowns and border closures [5,6,7,8]. Along with countries implementing lockdowns, home-healthcare services were also optimized in order to cater to the needs of COVID-19 patients at their homes in case they either did not require hospitalization or could not be admitted to hospitals due to a lack of patient beds or other vital medical facilities [9,10]. Multi-objective home-healthcare services involving the use of artificial-intelligence models were introduced, ensuring patient availability and convenience [9]. Furthermore, home-healthcare supply-chain frameworks have also been introduced based on programming models that aid patients in selecting pharmacies, enhance the selection and routing of nurses and also help caregivers connect with patients in a timely manner [10]. Despite these protective measures, the coronavirus continued to spread and harm individuals including children, the elderly and people with medical conditions such as cancer, diabetes and respiratory diseases, who were also at the highest risk of contracting the infection [5,11,12,13]. Individuals who required access to routine medical services such as pregnant women and people with chronic conditions experienced mental health issues such as stress, depression and reduced emotional well-being [14,15,16,17]. The emotional well-being of parents and children also suffered due to the lack of educational and food resources and the enhanced stress and financial problems due to restricted and insufficient healthcare facilities, especially in less-developed countries [18,19,20,21,22]. Due to the overwhelming number of positive cases and the limited availability of medical devices, the pressure on healthcare workers (HCWs) has significantly increased and placed them at higher risk of contracting the contagion. Several studies have reported clinically significant depression, stress and decreased mental well-being in HCWs [23,24]. Due to these numerous harmful effects of the COVID-19 virus, there is a crucial need to develop and administer vaccines in order to eliminate this deadly pandemic [24,25].

The Coalition for Epidemic Preparedness Innovations (CEPI) has been cooperating with the World Health Organization (WHO) to aid the vaccine developers in successfully developing and deploying COVID-19 vaccines [26]. The rapid development of the COVID-19 vaccine was seen as a necessity to suppress the repeated infection waves and lower the mortality rate [27]. The COVID-19 vaccine-development efforts started in March 2020 with the first vaccine candidate entering human trials on 16 March 2020. On 4 January 2021, the United Kingdom (U.K.) became the first country to administer a COVID-19 vaccine, which was manufactured by AstraZeneca in association with Oxford University [28]. Soon, other countries started their own vaccine campaigns. For example, China administered vaccines developed by home manufacturers such as Sinopharm, Sinovac and Cansino Biologica; Russia administered its vaccine known as Sputnik V; and the United States (U.S.) has been using vaccines developed by home manufacturers including Pfizer-BioNTech, Moderna, and Johnson and Johnson [28]. The rapid and sustained administration of the COVID-19 vaccine is critical for the world to return to the pre-pandemic normalcy. As of November 2021, one hundred and seventy-five vaccines are in clinical trials, forty-one have been approved and reached the final testing phase, and seventy-five are undergoing animal trials. Although the current progress in vaccine development and administration is encouraging, social-distancing and face-mask mandates are still in place in various regions to counteract the infection [29,30,31]. This is because, to be effective, the COVID-19 vaccines must be administered to the majority of the world population [32]. However, variations in vaccine acceptance and hesitancy have been observed in different groups across the world [33]. This means that the world, at large, is at risk of yet another pandemic as new SARS-CoV-2 variants continue to emerge.

At this time, there is a need to understand how and why COVID-19 vaccine-acceptance and hesitancy rates differ in various parts of the world, so institutions such as governments and non-governmental organizations can formulate strategies to promote COVID-19 vaccines in their respective regions. To this end, researchers have been studying COVID-19 vaccine acceptance in specific regions of the world [34,35]. A few systematic reviews analyzing the worldwide vaccine-hesitancy and acceptance rates have also recently surfaced [36,37]. However, there has not been a systematic and comparative study of the variations in vaccine acceptance across countries. In addition, a study of the social and behavioral factors responsible for the significant differences in acceptance rates is needed in order to understand the reasons behind acceptance and hesitancy of the COVID-19 vaccine in different world regions. A systematic review that statistically analyzes the differences in COVID-19 vaccine-acceptance rates of different countries, along with the reasons behind these rates, can support such an endeavor. It can also aid in the identification of fundamental social and behavioral factors that are ultimately responsible for vaccine acceptance and hesitancy. Researchers can use such results to conduct additional research to understand the correlations between COVID-19 vaccine acceptance and other factors in different contexts. Furthermore, government agencies and non-governmental organizations can use this knowledge to devise strategies to accelerate vaccine acceptance all over the world and put an end to this pandemic. In essence, our research questions are as follows:

- How do the COVID-19 vaccine-acceptance rates differ among different countries?
- How do the COVID-19 vaccine-acceptance rates differ among different continents?
- What social and behavioral factors are responsible for country-level differences in COVID-19 vaccine-acceptance rates?

In accordance with the research questions, the following are the highlights of our research:

- A systematic and comparative study about the variations in COVID-19 vaccine-acceptance rates across different countries and continents.
- Statistical analysis of the reported COVID-19 vaccine-acceptance rates.
- Determination of associated social and behavioral factors in relation to COVID-19 vaccine acceptance and vaccine hesitancy.

## 2. Materials and Methods

We report this systematic review according to the Preferred Reporting Items for Systematic Reviews and Meta-Analyses (PRISMA) guidelines [38] as well as the essential statement recommendations [38]. This section comprises the information sources and the search strategies that were employed for obtaining the selected studies. This is followed by the study-selection process and the eligibility criteria including the inclusion and exclusion criteria. The section is concluded with a statistical analysis that was executed to determine if there is a significant difference between the reported vaccine-acceptance rates of different countries.

### 2.1. Information Sources and Search Strategy

Relevant publications were identified through electronic searches in four databases: PubMed, Web of Science, IEEE Xplore and Science Direct. Articles were screened within the time period from 1 January 2020 to 31 July 2021. The databases were searched using the following keywords: “COVID-19”, “vaccine acceptance”, “vaccine hesitancy”, and “associated factors”. The Boolean operator ‘AND’ was utilized for searching the databases with the mentioned keywords.

### 2.2. Study Selection

The authors first screened article titles and abstracts to remove all the duplicates. The remaining articles were then independently reviewed, which involved reading article titles, abstracts and methods. The articles that did not meet our inclusion criteria, which were summarized as PVAF (Peer-reviewed (P), about the COVID-19 vaccine (V), reports the acceptance rate (A), provides social and/or behavioral factors (F)), were removed. The authors then met to compare their article selections and resolve disagreements through productive discussions. Once the article selection was finalized, the full text of each was independently reviewed by the authors.

### 2.3. Eligibility Criteria

Peer-reviewed publications that reported COVID-19 vaccine acceptance and the associated social and behavioral factors responsible for vaccine hesitancy were considered for inclusion. Furthermore, only studies published in English met the eligibility criteria. The exclusion criteria excluded articles that were pre-prints or unpublished as well as articles in a language other than English.

### 2.4. Statistical Analysis

A nonparametric one-way ANOVA was performed using the IBM Statistical Package for Social Sciences (SPSS) Version 26.0 on a Windows 10 machine to investigate whether a statistically significant difference existed between the reported vaccine-acceptance rates of different countries. This was followed by the application of the Kruskal–Wallis H test to conduct pairwise comparisons between the vaccine-acceptance rates of all the included countries.

## 3. Results

**Figure 1:**
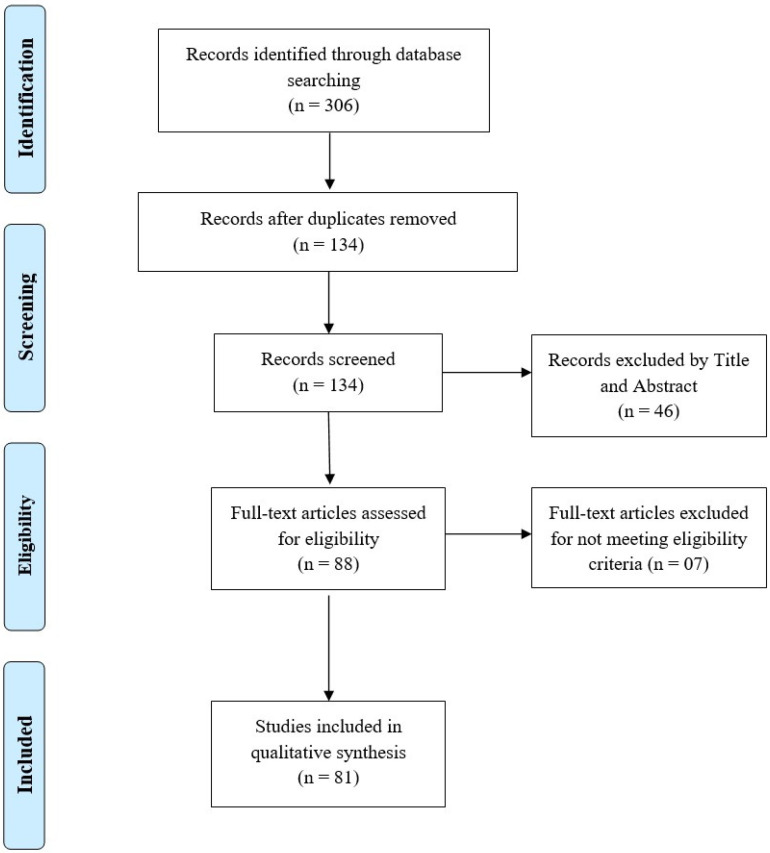
Study identification PRISMA flowchart.

The initial electronic search strategy returned 306 research articles, as demonstrated by Figure 1. Only peer-reviewed research articles published in journals were taken into consideration. After the removal of the duplicates, 134 research articles remained; of these, 46 publications were excluded after title and abstract reviews because they did not report COVID-19 vaccine-acceptance and hesitancy rates, and/or the associated social and behavioral factors. The remaining 88 articles then underwent elaborate reviews in which each section of every research article was thoroughly analyzed. Following this, 81 relevant research articles were selected for inclusion in this systematic review. Table 1 summarizes the selected research articles along with their publication year, number and type of participants, COVID-19 vaccine-acceptance rates, and acceptance and hesitancy factors.

### 3.1. Characteristics of the Papers Included

**Figure 2:**
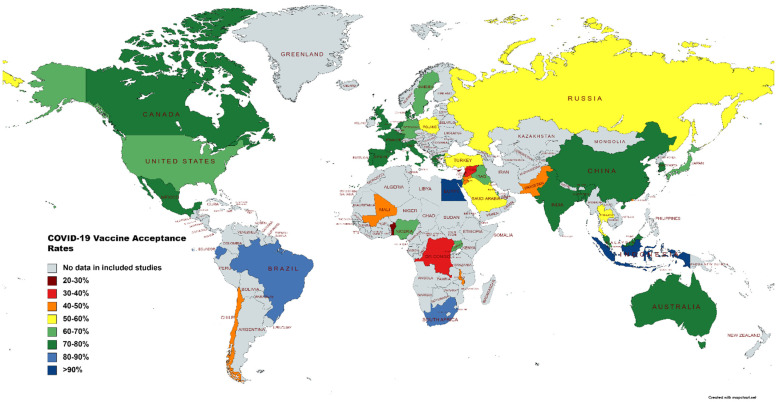
Map illustrating vaccine-acceptance rates worldwide.

81 papers were selected for this systematic review, with the most papers being from China (*n* = 12), followed by Italy (*n* = 8) and then the U.S (*n* = 8). Also included in this review were studies conducted in Saudi Arabia (*n* = 5), France (*n* = 4), Hong Kong (*n* = 4), Turkey (*n* = 4) and the U.K. (*n* = 4). While the majority of the studies were published in 2020, the most recent paper was published in June 2021. Six studies involved more than one country. Neumann-Böhme et al. [95] published a study spanning seven European countries, Lazarus et al. [34] focused on nineteen countries, and the research of Bono et al. [35] was conducted in nine countries. Taylor et al. [110], Salali and Uysal [98] and Sallam et al. [55] conducted their studies in two countries each. All of the studies focused on adults, with the exception of Zhang et al. [74], who worked with children below 18 years of age. Among the included studies, 57 surveys included the general population and 16 included HCWs. Three studies focused on multiple groups including the general population, HCWs and healthcare college students [68,78,88]. Two studies focused solely on dentists, dental surgeons and dental students [104]. Lazarus et al. [34] had the largest sample size (*n* = 13,426), while Mascarenhas et al. [104] had the smallest (*n* = 248). A total of fifty countries that reported their COVID-19 vaccine-acceptance rates are highlighted in Figure 2.

### 3.2. Rates of COVID-19 Vaccine Acceptance

**Figure 3:**
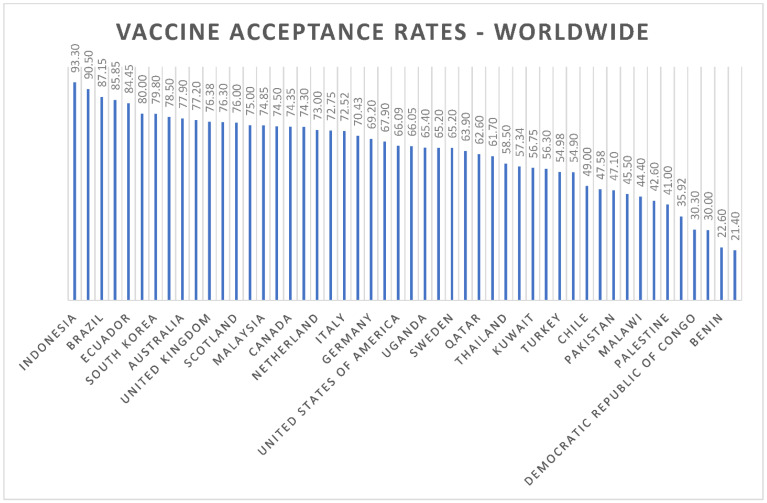
Worldwide COVID-19 vaccine-acceptance rates.

**Figure 4:**
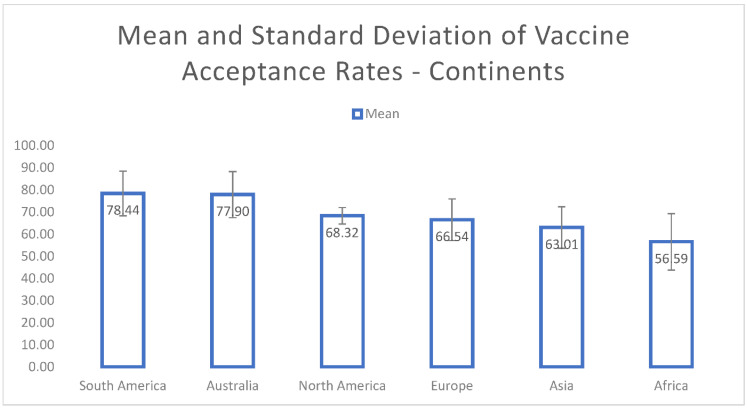
Mean and standard deviation of COVID-19 vaccine-acceptance rates for continents.

The mean COVID-19 vaccine-acceptance rates of countries are illustrated in Figure 3. Indonesia (93.30%) had the highest mean vaccine-acceptance rate followed by Egypt (90.50%), Brazil (87.15%), South Africa (85.85%), Ecuador (84.45%) and Denmark (80%). The means and standard deviations for the continents are shown in Figure 4. We found that the highest mean vaccine-acceptance rate was reported by South America (78.44%), whereas the lowest mean vaccine-acceptance rate was reported in Africa (56.59%).

Among adults from the general population, the highest vaccine-acceptance rates were reported in Ecuador (97%), Malaysia (94.3%) and Indonesia (93.3%), and the lowest rate was reported in Lebanon (21.40%). In the healthcare workers (HCWs) category, general HCWs in China (86.20%) and nurses in Italy (91.50%) had the highest acceptance rates. The HCWs in the Democratic Republic of Congo had the lowest acceptance rate (27.70%). Among the patients with chronic diseases, those with rheumatic disease in Turkey showed a vaccine-acceptance rate of 29.2%, adolescent cancer survivors in the U.S. had an acceptance rate of 63%, and patients with type-two diabetes mellitus in Italy reported an acceptance rate of 85.80%. One study based in China reported a 77.4% vaccine-acceptance rate among pregnant women.

### 3.3. Demographic Factors Influencing COVID-19 Vaccine-Acceptance Rates

#### 3.3.1. North America

The mean COVID-19 vaccine-acceptance rates in the North American countries, particularly the United States (U.S.), Mexico and Canada, were in the range of 56% and 75%. The general-population participants who were recruited in the U.S., Canada and Mexico by Lazarus et al. [34] exhibited COVID-19 vaccine-acceptance rates of 76.3%, 75.4% and 68.7% respectively. Social influences, including an employer’s advice to get vaccinated, and behavioral factors, such as an accelerated trust in government directives about the significance of getting vaccinated against COVID-19, played a major role in achieving enhanced vaccine-acceptance rates.

Lower vaccine-acceptance rates were found in studies by Mascarenhas et al. [104] and Viswaanth et al. [105], where 56% and 65% individuals in the United States, respectively, were found to be willing to receive the COVID-19 vaccine. The lowered trust in public-health experts and the perceived risk of receiving the COVID-19 vaccine were the major behavioral factors responsible for low vaccine-acceptance rates among dental students in the U.S. [104]. Social factors including a higher exposure to different social-media platforms combined with the behavioral perceptions about the risks of vaccines were the major factors responsible for lower vaccine-acceptance rates in U.S. adults [105]. The educational background was mentioned as the major socio-demographic factor responsible for low vaccine acceptance (63%) among adolescent and young-adult cancer survivors in the U.S. [103]. The behavioral determinants pertaining to the hesitancy towards the COVID-19 vaccine in the U.S. and Canada comprised of misconceptions and misinformation surrounding the efficacy and side effects of the vaccine [106,107]. Testing time and the requirement of a second dose also led to decreased acceptance rates [107].

Overall, social dynamics in the United States including low educational levels and awareness, race, younger age, gender, employment directives and lack of trust in government institutions led to lowered vaccine-acceptance rates [106,108,109]. Furthermore, in the U.S. and Canada, many individuals mentioned relying upon their natural immunity instead of receiving the vaccine [110].

#### 3.3.2. South America

The COVID-19 vaccine-acceptance rate in South America ranged from 49% to 97%. The lowest vaccine-acceptance rate was found in Chile where 49% of the participants were willing to receive a vaccine [116]. The low vaccine-acceptance rate in Chile was due to the lack of government-initiated vaccine-awareness campaigns and the perceived vaccine side effects among the general public [116].

On the contrary, the highest vaccine-acceptance rate was observed in Ecuador where 97% of the surveyed adults were eager to get vaccinated [117]. Ecuador was characterized by an accelerated trust in government institutions and higher vaccine-related education. Furthermore, people showed a willingness to pay for the vaccine [117].

#### 3.3.3. Europe

The vaccine-acceptance rate in Europe ranged from 27% to 91.5%. Neumann-Bohme et al. [95] studied seven European countries, which included Denmark, the U.K., Portugal, the Netherlands, Germany, France, and Italy. Vaccine hesitancy was related to mistrust in a vaccine that had been prepared in a very short amount of time. Eight studies were conducted in Italy, with the lowest vaccine-acceptance rate reported as 27% [80] and the highest as 91.5% [83]. Another study [80] surveyed parents about vaccinating their children and found that the uncertainty about vaccine safety was the major factor for vaccine rejection.

Two studies carried out in Scotland demonstrated a higher vaccine-acceptance rate; the earlier study reported an acceptance rate of 74% and the latter reported 78% [89]. Williams et al. [89] associated socio-demographic factors such as higher income and social status with a higher intention to receive the vaccine. Four separate studies focusing on France determined the vaccine-acceptance rate to range from 71.2% [92] to 77.6% [99]. Gagneux-Brunon et al. [93] analyzed vaccine acceptance among HCWs in France and found that nurses were more hesitant towards getting vaccinated. Detoc et al. [99] assessed the general population in France and related vaccine hesitancy to the perceived risks. Four studies were also carried out in the U.K. and reported vaccine acceptance ranging from 64% [94] to 89.10% [97]. Racial and ethnic minorities and low-income households were most prominently linked with vaccine hesitancy in the U.K. [97]. Turkey had a lower vaccine-acceptance rate ranging from 34.6% [88] to 51.6% [86]. Yigit et al. [87] focused on HCWs in Turkey and İkiışık et al. [86] targeted the general population. Both studies found age to be a factor in vaccine hesitancy; specifically, a younger age was associated with a greater vaccine hesitancy. Only one study from Cyprus was considered in this review, and it showed a very low acceptance rate of 30% [90]. This was mainly due to the fear of side effects related to the vaccine.

Social and behavioral factors such as anxiety, government enforcement and risk perception proved to be hindrances to vaccine acceptance in countries such as the U.K. and Turkey [98]. Vaccine hesitancy was also associated with negative beliefs including mistrust, conspiracy theories and negative support by the health professionals [96]. The female sex and confidence in vaccine efficacy were related to higher vaccine acceptance [83]. Fear of the unknown scientific results led to a very low vaccine-acceptance rate among patients with rheumatic diseases in Turkey [88].

#### 3.3.4. Australia

Two studies carried out in Australia have been considered in this review. Both reported high vaccine-acceptance rates of 75% to 80%. Rhodes et al. [112] studied parents of school-going children (*n* = 2018). Researchers indicated that knowledge about COVID-19 and older age were key factors in vaccine acceptance. Seale et al. reported that family support greatly increased vaccine acceptance [111].

#### 3.3.5. Asia

The vaccine-acceptance rates for Asia, as reported in the included studies, ranged from 21.40% to 94.30%. China, where the largest number of studies were reported, ranged in acceptance rates from 36.40% to 91.30%. Low vaccine-acceptance rates in China were found to be prevalent among college students and children below 18 years of age. The general population, HCWs and pregnant women exhibited high vaccine-acceptance rates. The factors associated with higher acceptance rates included an enhanced trust in government initiatives, an employer’s advice regarding vaccination, and valuing a health professional’s recommendation due to being at higher risk of infection [39,40,41,46,47,72,73]. The factors associated with lower acceptance rates were a lack of confidence in the effectiveness of the vaccine, its side effects, and a lack of knowledge or misinformation among the participants regarding the potential harms of the vaccines [42,43,74].

Saudi Arabia reported acceptance rates ranging from 48.00% to 64.70%. Socio-demographic factors such as high income, being married, and being a resident of a major city were negatively correlated with vaccine acceptance [77]. On the contrary, factors associated with vaccine acceptance included positive information and awareness regarding the effectiveness of vaccines and the previous uptake of influenza vaccine. Government strategies and initiatives including advertisements about the benefits of the COVID-19 vaccine and targeted health education were used for spreading positive information and awareness regarding the effectiveness of the COVID-19 vaccine [77]. Uncertainty surrounding the safety and efficacy of the vaccines increased vaccine hesitancy among participants [48,49,50,51].

Low vaccine-acceptance rates in Kuwait and Jordan were reported due to behavioral and social factors including low confidence in healthcare professionals, belief in conspiracy theories such as that vaccines lead to infertility, and misinformation such as that the vaccine alters one’s genes, that it contains a tracking device, or that it is unsafe [52,53,54,55,56,57].

The higher acceptance rate in Malaysia was due to socio-economic factors such as higher education levels and self-awareness [79]. In Hong Kong, lower acceptance rates were associated with low education levels, a lack of vaccine-awareness initiatives by the government, and a history of past pandemic sufferings [58,60,75]. The safety of the vaccines was also a major issue and it negatively impacted smokers’ and chronic-disease patients’ decisions to get vaccinated [59].

The higher acceptance rates reported in the rest of the Asian countries were due to self-awareness among participants. Participants of older age groups were more willing to get vaccinated as well as those who trusted their governments, employers and healthcare professionals. The participants who showed hesitancy towards vaccines were more concerned about their side effects than their benefits. This was usually observed in low-income and low-education groups.

#### 3.3.6. Africa

The vaccine-acceptance rates in Africa ranged from 27.70% to 90.50%. The highest vaccine-acceptance rate among African countries was exhibited by South Africa where 90.50% participants were willing to get vaccinated [113]. Socio-demographic factors including higher income and government initiatives, such as campaigns releasing information about the vaccination in a timely manner and public service announcements on cellular networks about getting vaccinated, played a major role in the higher acceptance of COVID-19 vaccines [113]. Other factors associated with vaccine acceptance included positive information and awareness about the benefits of vaccines and the previous uptake of influenza vaccine [34].

The factors associated with higher acceptance rates in other African countries were higher education levels that led to higher awareness and knowledge about the advantages of getting vaccinated, and the government and employers making vaccination mandatory [35]. In countries such as Democratic Republic of Congo, where a low vaccine-acceptance rate of 27.70% was reported, factors such being of a younger age and lacking knowledge about COVID-19 vaccines and their benefits led people to believe that it was unsafe to get vaccinated [115]. Participants with a history of chronic diseases were also linked with vaccine hesitancy.

### 3.4. Comparisons between Countries

The one-way ANOVA revealed that there was a statistically significant difference in the COVID-19 vaccine-acceptance rates of at least two countries (H (49) = 75.302, *p* = 0.009*).

**Figure 5:**
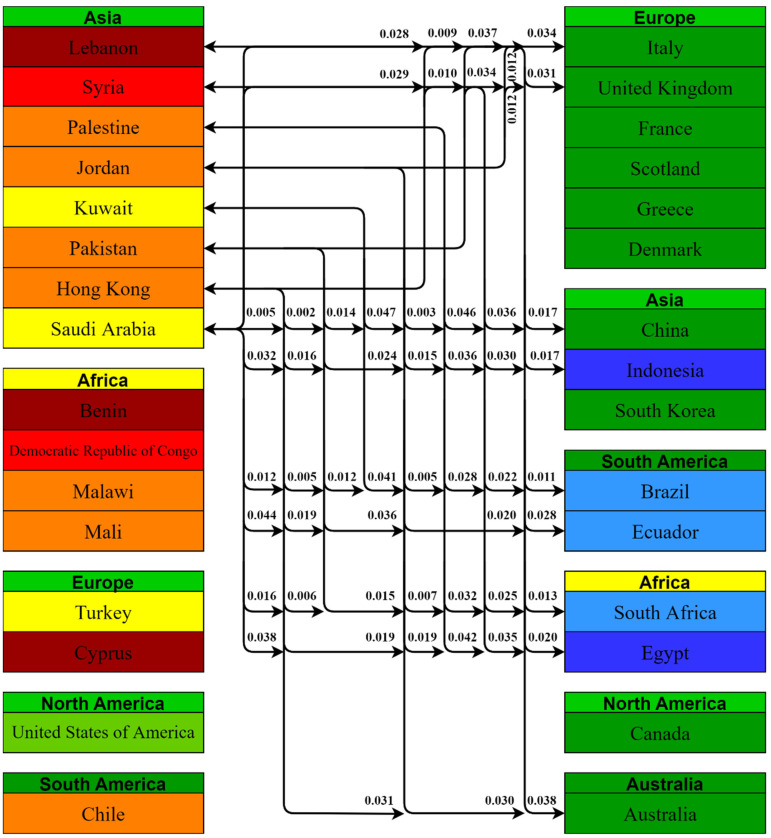
Pairwise comparison between Asian countries having lesser acceptance rates with the rest of the world and their *p* values.

**Figure 6:**
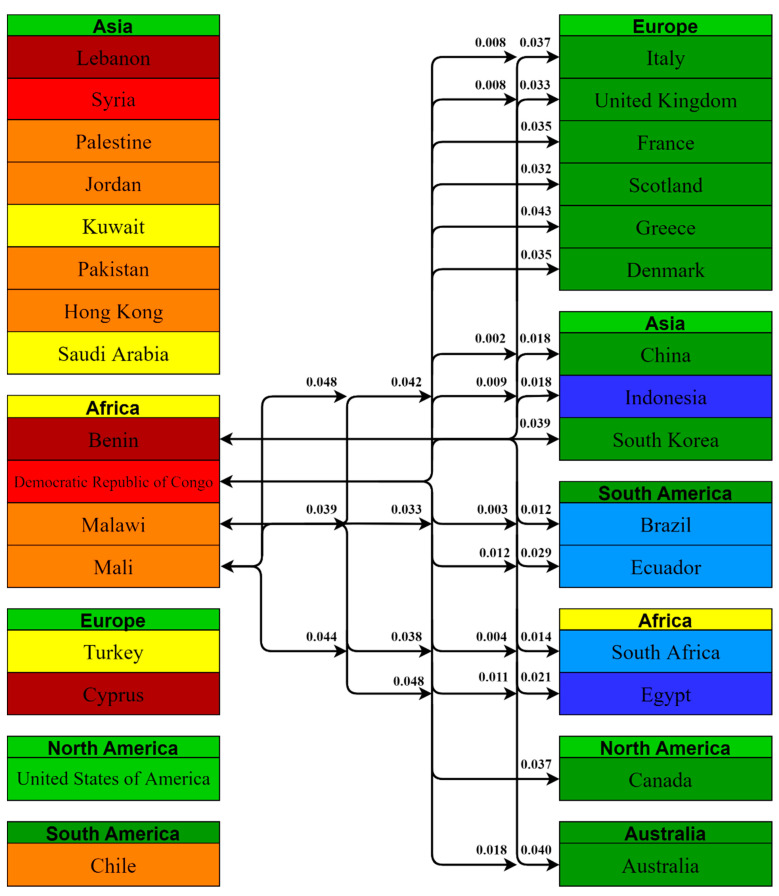
Pairwise comparison between African countries having lesser acceptance rates with the rest of the world and their *p* values.

**Figure 7:**
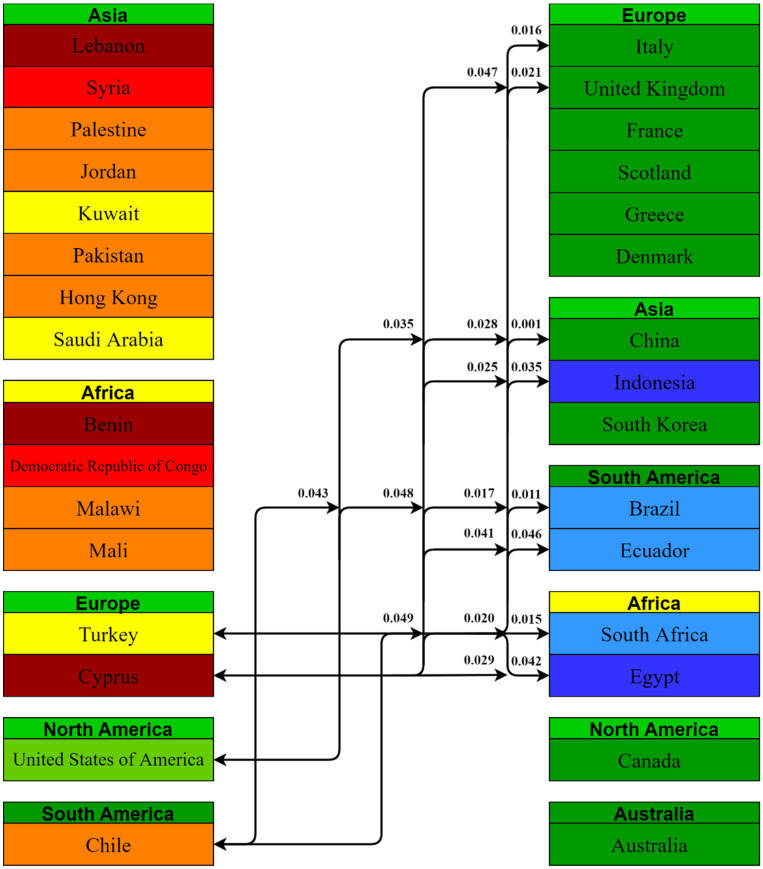
Pairwise comparison between European, North and South American countries having lesser acceptance rates with the rest of the world and their *p* values.

Using the Kruskal–Wallis H Test, a total of 1225 comparisons were obtained between the 50 countries included in this study, among which only 105 comparisons had a value of *p* < 0.05, i.e., 31 countries had statistically significant differences between some of their vaccine-acceptance rates. We grouped the countries by their continents and the statistically significant *p* values are presented in Figure 5, Figure 6 and Figure 7.

Asian countries with lower acceptance rates had 55 statistically significant comparisons; 15 of these comparisons were with other Asian countries, 14 with African countries, 13 with South American countries, 10 with European countries and 3 with Australia. Lebanon, Jordan, and Hong Kong were involved in nine statistically significant (*p* < 0.05) comparisons.

African countries with lower acceptance rates had statistically significant comparisons with 31 countries; 8 comparisons were with European countries, 7 with other Asian countries, 7 with other African countries, and 6 with South American countries. Democratic Republic of Congo had the most comparisons, i.e., 15, with a value of *p* < 0.05, which is the highest among the included countries.

European countries with lower acceptance rates had statistically significant comparisons with 15 countries; Asian, South American and African countries each had 4 comparisons whereas 3 comparisons were with European countries.

North and South American countries with comparatively lower acceptance rates had two comparisons each with other countries of the world.

## 4. Discussion

The willingness to accept a vaccine is known to be triggered by three parameters: complacency, confidence and convenience [33]. Complacency refers to the assumption that the risk of contracting a particular disease is low and hence, that vaccination is inessential and avoidable [36,118]. Confidence is one’s level of trust and conviction in the welfare and usefulness of vaccination. Convenience involves the comfort provided to the population in terms of vaccine accessibility, affordability and supply [36].

With the start of the spread of the coronavirus, the rapid development of vaccines began and ultimately, the deployment of vaccines against COVID-19 was witnessed. However, in order to build herd immunity and ensure that mortality rates are lowered, worldwide vaccine acceptance is necessary [119]. Vaccine hesitancy has been observed to be a major hindrance in the global efforts to curb the spread of the coronavirus and is primarily due to social and behavioral influences [120]. Hence, the goals of this systematic review comprised of assessing the differences in COVID-19 vaccine-acceptance rates among different countries and among different continents. Furthermore, this systematic review aimed to determine the social and behavioral factors that form the basis for significant differences between COVID-19 vaccine acceptance among different countries and continents. Our findings support the aims of this systematic review and the results demonstrated differences between COVID-19 vaccine-acceptance rates among different countries. Indonesia reported the highest mean vaccine-acceptance rate (93.30%), whereas Lebanon demonstrated the lowest mean acceptance rate of 21.40%. Similarly, the findings of this systematic review demonstrated the differences between COVID-19 vaccine-acceptance rates among different continents, with South America exhibiting the highest mean acceptance rate of 78.44%, while Africa reported the lowest rate of 56.59%. Certain social and behavioral factors that differed between different countries and continents were responsible for the significant differences between COVID-19 vaccine-acceptance rates.

Significant differences were found between the vaccine-acceptance rates of North America and Europe pertaining to the social and behavioral factors. Lower vaccine-acceptance rates were found in Europe, particularly in countries including the U.K and Denmark, due to reasons such as low income, cultural influences, political beliefs and conspiracy theories relating to the negative attitude of medical professionals [95,96,97]. Hence, in order to enhance the vaccine-acceptance rates in Europe, government institutions should implement strategies that help to eliminate political differences and cultural influences. Community groups can also hold seminars that help individuals not to believe in conspiracy theories and persuade them about the potential benefits of receiving the COVID-19 vaccine [121]. Significant differences between vaccine-acceptance rates were also found between North America and Asia. Lower vaccine-acceptance rates in Asia were due to factors including low educational levels, a lack of awareness regarding the potential benefits of vaccination, low levels of income and a lack of confidence among the individuals [39,40,41,42,43]. Concerns regarding virus mutation were also prevalent in Asia [44].

Significant differences were found between the vaccine-acceptance rates in South America and Asia. Lower vaccine-acceptance rates in Asia were due to factors including a lack of adequate knowledge and awareness, low educational levels, an absence of influenza vaccine history and the prevalence of conspiracy theories including perceived risks about vaccination leading to infertility [50,51,52,53,54,55,56,59]. Moreover, a preference for natural immunity and misinformation on social-media platforms also contributed to low vaccine-acceptance rates in Asia [70]. Significant differences between vaccine-acceptance rates were found in Europe and Asia. The low vaccine-acceptance rate in Asia was due to low levels of education and awareness, the prevalence of conspiracy theories relating to infertility and side effects, a poor influenza vaccination history and a lack of confidence in healthcare professionals [60,61,62,63,64]. Moreover, people in Asia preferred their natural immunity over vaccines and social-media influence also played a role in creating misinformation and spreading conspiracy theories among the population [70].

Significant differences were observed between COVID-19 vaccine-acceptance rates in Australia and Asia. The reasons for low vaccine-acceptance rates in Asia were primarily due to low levels of education and awareness, the prevalence of conspiracy theories relating to infertility and side effects, a poor influenza vaccination history and a lack of confidence in healthcare professionals [65,66,67,68,69,72,73,74,75]. Hence, there is a need for government institutions and non-governmental organizations in Asia to devise and implement campaigns that help to elevate the awareness level relating to the administration of the COVID-19 vaccine. Enhanced awareness levels will also boost the confidence among individuals and the perceived risks associated with virus mutation and vaccine safety will also decrease [122]. Moreover, information-technology companies in Asia should focus on eliminating incorrect information about the COVID-19 vaccine on different social-media platforms and instead should ensure the creation of websites and webpages that indicate the advantages of receiving the vaccine [123].

The findings of this systematic review helped to identify the social and behavioral factors responsible for the significant differences between vaccine-acceptance rates that were observed in South America and Africa. Africa reported lower vaccine-acceptance rates as compared to South America due to individuals having a history of vaccine refusal, including the influenza vaccine [34,35]. A lack of knowledge and adequate awareness regarding the benefits of vaccination also played a major role in the lower vaccine acceptance in Africa [115].

Significant differences in vaccine-acceptance rates were found in Africa and Asia. Africa reported lower COVID-19 vaccine-acceptance rates primarily due to the major factor of vaccine refusal [34,35]. People in Africa reported a history of poor influenza vaccination due to fewer awareness programs initiated by the government and as a result they also showed resistance towards the COVID-19 vaccine. Moreover, high vaccine refusal was also due to the increased illiteracy rate in Africa as compared to Asia [115]. Similar differences were also found between Europe and Africa. Africa reported low vaccine-acceptance rates due to individuals exhibiting poor influenza vaccination history and a history of vaccine refusal due to low educational levels [34,35]. Hence, government institutions and non-government establishments in Africa should work towards drafting and implementing strategies that will help to gain the trust of individuals and accelerate vaccine acceptance.

## 5. Conclusions

COVID-19 vaccine-acceptance rates differ according to the countries and continents of the world. The majority of the studies included in this systematic review reported COVID-19 vaccine-acceptance rates of >60%. However, the lowest rate of 21.40% was reported by a study analyzing vaccine acceptance in Lebanon. The mean COVID-19 vaccine-acceptance rate among continents showed that South America had a greater population willing to become vaccinated against coronavirus. On the contrary, African countries reported significantly lower acceptance rates and hence, Africa has the lowest mean vaccine-acceptance rate.

The high and low COVID-19 vaccine-acceptance rates stemmed from various social and behavioral characteristics exhibited by the participants in the studies included in this systematic review. High COVID-19 vaccine-acceptance rates in South America, Australia and Europe emerged due to certain factors such as the increased trust of individuals in government health policies and the efficient strategies formulated by the policy makers, leading to enhanced awareness about the benefits of getting vaccinated against COVID-19. The social and behavioral factors that gave rise to significantly low vaccine-acceptance rates, particularly in Asia and Africa, comprised of low levels of education, awareness and inefficient efforts and initiatives by the government that led to mistrust and perceived threats about the COVID-19 vaccine among the population. Furthermore, a poor history of influenza vaccination, conspiracy theories relating to infertility and misinformation about the COVID-19 vaccine on social-media platforms also resulted in vaccine hesitancy. One limitation of this systematic review is that it only includes studies reporting COVID-19 vaccine-acceptance rates and associated factors of fifty countries.

Hence, future research can be carried out with additional countries. Moreover, in order to optimize global COVID-19 vaccine acceptance, the governments should ensure that people become educated about the benefits of COVID-19 vaccination and should implement policies that help to elevate the awareness among the population. Moreover, the necessary elimination of conspiracy theories and misinformation regarding vaccination should also be ensured. This will ultimately lead to high COVID-19 vaccine acceptance all over the world and will aid in fighting and putting an end to this pandemic.

## Figures and Tables

**Table 1:** Summary of the studies included in the systematic review based on the vaccine-acceptance rates and their associated social and behavioral factors. AF: acceptance factors; HF: hesitancy factors; HCWs: healthcare workers.

Continent	Year	Authors	Number of Participants	Type of Participant Population	Country	COVID-19 Vaccine-Acceptance Rate (%)	Associated Factors
Asia, Africa, Europe, North America, South America	2021	Lazarus et al. [34]	13,426	General Population	Brazil, Canada, China, Ecuador, France, Germany, Italy, India, Mexico, Nigeria, Poland, Russia, Singapore, South Africa, South Korea, Spain, Sweden, United Kingdom and United States	85.36%, 68.74%, 88.62%, 71.93%, 58.89%, 68.42%, 70.79%, 74.53%, 76.25%, 65.22%, 56.31%, 54.85%, 67.94%, 81.58%, 79.79%, 74.33%, 65.23%, 71.48%, 75.42%, respectively.	AF: Trust in government institutions and employers’ advice played major roles in enhancing vaccine-acceptance levels. HF: Men were less likely to accept a vaccine and individuals with lower income demonstrated vaccine hesitancy.
Asia, Africa, South America	2021	Bono et al. [35]	10,183	General population	Brazil, Malaysia, Thailand, Bangladesh, Democratic Republic of Congo, Benin, Uganda, Malawi and Mali	94.2%, 78.6%, 87.3%, 89.6%, 59.4%, 48.4%%, 88.8%, 61.7%, 74.5%, respectively.	AF: Social, medical and behavioral factors including knowledge relating to COVID-19, income, age, gender and chronic diseases were linked to vaccine-acceptance rates. HF: Perceived vaccine threats were linked to vaccine hesitancy.
Asia	2021	Wang et al. [39]	7381	Adults (Above 18 years)	China	75.3%	AF: Adults who had previously received the influenza vaccine, individuals who were older, were nonmedical personnel and had high educational levels demonstrated high vaccine acceptance. HF: Vaccine hesitancy due to the fear of side effects.
	2021	Xu et al. [40]	1051	Adults (Healthcare workers)	China	86.2%	AF: Accepted the vaccine after studying scientific literature and being encouraged by family members, friends, colleagues, experts and news media. HF: Request by employers and concern for vaccine safety was associated with vaccine hesitance.
	2021	Liu et al. [41]	2377	Adults	China	82.25%	AF: Older age, medical insurance and vaccine safety for both paid and free vaccines. Factors relating high income, perceived benefits of vaccine were responsible for vaccine acceptance. HF: Young age, low educational levels and concerns about vaccine safety and side effects led towards vaccine hesitancy.
	2021	Gan et al. [42]	1009	Adults (General Population)	China	60.40%	AF: Middle aged people with higher education, those with a past influenza vaccination history and perceived effectiveness of the COVID-19 vaccine resulted in vaccine acceptance. HF: Lower education levels, different occupations and lack of influenza vaccination resulted in vaccine hesitancy.
	2021	Walker et al. [43]	330	College Students	China	36.4%	AF: Perceived vaccine benefits resulted in vaccine acceptance. HF: Vaccine hesitancy among international college students was due to perception of barriers, lack of knowledge, place of residence and program of study.
	2021	Sun et al. [44]	505	Healthcare Workers	China	76.63%	AF: Understanding the benefits of the vaccine, perceived risks of COVID-19, living with elderly individuals and history of influenza vaccination resulted in vaccine acceptance. HF: Major contributors to vaccine hesitancy were perceptions about vaccine safety, effectiveness and concerns relating to rapid mutation of the virus.
	2021	Tao et al. [45]	1392	Pregnant Women	China	77.4%	AF: High level of perceived susceptibility of COVID-19, significant vaccine information, high level of perceived vaccine benefits, living in western region and young age resulted in vaccine acceptance. HF: Low level of education, older age, and perceived risks of vaccine side effects led to vaccine hesitancy.
	2021	Chen et al. [46]	3195	Adults	China	83.8%	AF: Belief that the vaccine would be beneficial to health and enhanced trust in government institutions and health experts resulted in vaccine acceptance. HF: Lack of confidence, risks associated with vaccine and awareness frequency was the main contributors affecting the intention of receiving the COVID-19 vaccine.
	2021	Han et al. [47]	2126	Migrant population	Shanghai, China	89.1%	AF: Higher acceptance rates were demonstrated by younger individuals, families with three to four members, and those with higher education and income. HF: Vaccine hesitancy contributors included lack of information and confidence and willingness to pay for the vaccine.
	2021	Fayed et al. [48]	980	Adults (General Population)	Saudi Arabia	59.5%	AF: Demographic characteristics and the willingness to be vaccinated against the seasonal influenza. HF: Lack of trust in government institutions and perceived risks about vaccine side effects and safety.
	2021	Qattan et al. [49]	673	Healthcare Workers	Saudi Arabia	50.52%	AF: Being a male healthcare worker, perceiving an elevated risk of infection and adhering to the compulsory vaccination requirement were contributors for high vaccine-acceptance rates. HF: Young age, female gender, and HCWs residing in the south showed vaccine hesitancy.
	2021	Alfageeh et al. [50]	2137	Adults	Saudi Arabia	48%	AF: Individuals residing in the southern region, past influenza vaccination, perceived risks of contracting the coronavirus and belief in mandatory vaccination were responsible for vaccine acceptance. HF: Social and behavioral factors pertaining to vaccine safety, history of vaccine refusal, attitudes towards seasonal influenza vaccination made up for the vaccine hesitancy.
	2021	Alshahrani et al. [51]	Not reported	General population	Saudi Arabia	64%	AF: Factors associated with vaccine acceptance included vaccine information and awareness, perceptions towards vaccine effectiveness and previous uptake of influenza vaccine. HF: Misinformation relating to the side effects of the vaccine increased vaccine hesitancy.
	2021	AlAwadhi et al. [52]	7241	Adults	Kuwait	67%	AF: Increased agreement with containment policies, high confidence in medical professionals and high awareness regarding the benefits of the vaccine increased acceptance rates. HF: Behavioral and social factors such as having low confidence in doctors, perceived fear and worries were evaluated to the reasons behind vaccine hesitancy.
	2021	Alqudeimat Y et al. [53]	2368	Adults (above 21 years)	Kuwait	53.1%	AF: Past influenza vaccination history, male gender, and increased perceptions about the benefits of the vaccine improved acceptance rates. HF: Safety concerns regarding the vaccine and lack of influenza vaccine history increased vaccine hesitancy chances.
	2021	Al-Sanafi et al. [54]	1019	Adults (Healthcare workers)	Kuwait	83.3%	AF: High levels of trust and confidence in government institutions and health systems resulted in high vaccine-acceptance rates. HF: Vaccine hesitancy was linked to social and behavioral factors such as conspiracy theories, gender and lower educational levels.
	2021	Sallam et al. [55]	771; 2173	Adults (General Population)	Kuwait, Jordan	23.6%; 28.4%	HF: Vaccine conspiracy beliefs such as injection of microchips and vaccine administration leading to infertility. Vaccine hesitancy related to exposure to social-media platforms displaying negative information.
	2021	Qerem and Jarab [56]	1144	Adults (General Population)	Jordan	36.8%	HF: High refusal and hesitancy were due to concern regarding use of vaccines and lack of trust.
	2021	El-Elimat et al. [57]	3100	Adults (General population)	Jordan	62.6%	AF: Males and who took the influenza vaccine before demonstrated vaccine acceptance. Moreover, willingness to pay, and perceived benefits of the vaccine helped in increasing the acceptance rates. HF: Social and behavioral factors including female gender, lack of influenza vaccine history, less knowledge regarding vaccine safety and conspiracy theories behind the COVID-19 virus.
	2021	Yan et al. [58]	1255	Adults (General population)	Hong Kong	42%	AF: Vaccine acceptance associated with male gender, witnessing of previous pandemics, and government influence. HF: Individuals who were female, older aged and with lower educational levels were more likely to show hesitancy towards the vaccine.
	2021	Luk et al. [59]	1501	Adults	Hong Kong	45.3%	AF: Older people and individuals with chronic diseases demonstrated vaccine acceptance. HF: The most prevalent vaccine hesitancy factors included vaccine safety concerns, younger adults, those with chronic diseases and smokers. Furthermore, social factors comprised of inadequate knowledge and decelerated perceived risk of danger of not getting vaccinated.
	2021	Kwok et al. [60]	1205	Adults (Nurses)	Hong Kong	63.00%	AF: Younger age, more confidence in HCWs and past influenza vaccination history resulted in vaccine acceptance. HF: Lack of influenza vaccination, older individuals and less educational levels were responsible for vaccine hesitancy.
	2021	Machida et al. [61]	2956	General Population	Japan	62.1%	AF: Men who were aged 65 and above demonstrated vaccine acceptance. Individuals, who were married, were suffering from chronic diseases and had high educational levels exhibited high vaccine acceptance. HF: Psychological factors such as perceived side effects of vaccine and social factors including lower income groups, and adults aged 20–49 were hesitant to receive the COVID-19 vaccine.
	2021	Yoda and Katsuyama [62]	1100	Adults (General Population)	Japan	65.7%	AF: Willingness to be vaccinated was associated with social factors such as older age groups, rural residences and individuals with underlying medical conditions. HF: Vaccine hesitancy was related to male gender.
	2021	Chaudhary et al. [63]	423	General population	Pakistan	53%	AF: Healthy individuals with high income and educational backgrounds were more willing to get vaccinated. HF: Social and behavioral factors including lack of knowledge, perceptions of vaccine risks and perception of vaccine safety encapsulated the factors for low vaccine-acceptance rates.
	2021	Arshad et al. [64]	2158	Adults (General Population)	Pakistan	41.2%	AF: Willingness to pay for the vaccine developed by Sino Pharm resulted in vaccine acceptance. HF: Conspiracy beliefs strongly associated with vaccine rejection.
	2021	Mulla et al. [65]	462	Adults	Qatar	62.6%	AF: Social and behavioral factors including gender, having a postgraduate degree, government ruling on making vaccinations mandatory for travel and safety concerns. HF: Concerns regarding the rushed pace of development of a vaccine and its side effects, and the emergence of new variants of the coronavirus were responsible for vaccine hesitancy.
	2021	Abedin et al. [66]	3646	Adults	Bangladesh	74.6%	AF: Trust in health safety regulations and high confidence in country’s health system resulted in vaccine acceptance. HF: Vaccine hesitancy resulted in social factors such as low educational levels, health and clinical related factors including chronic diseases.
	2021	Al Halabi et al. [67]	579	Adults	Lebanon	21.4%	HF: Mainly females, married participants and those who had a general vaccine hesitancy comprised of the high percentage of people exhibiting low willingness to receive the vaccine.
	2021	Al-Metwali et al. [68]	1680	Healthcare workers, general population and health college students	Iraq	61.7%	AF: HCWs and individuals who had received the influenza vaccination in the past were more willing to get vaccinated. HF: Concerns with storage, perceived benefits, perceived barriers and less awareness about vaccination formed the basis for the factors leading to vaccine hesitancy.
	2021	Mohamad et al. [69]	3402	Adults	Syria	35.92%	HF: Factors including gender, age, not having children, rural residence, smoking and perceived risks of vaccine side effects and low educational levels were responsible for the poor vaccine-acceptance rate.
	2021	Rabi et al. [70]	639	Nurses	Palestine	41%	HF: Lack of knowledge pertaining to the vaccine, age, perceived risk of side effects and preference to natural immunity comprised of social and behavioral factors responsible for low vaccine-acceptance rate.
	2021	Zigron et al. [71]	506	Adults (Dentists and dental residents)	Israel	85%	AF: Increase in unemployment rate led towards enhanced vaccine acceptance. HF: Decreased unemployment rate resulted in less willingness to inoculate with the vaccine.
	2020	Lin et al. [72]	3541	Adults (General Population)	China	83.50%	AF: The willingness to pay for the vaccine was influenced by socio-economic factors, such as preference of domestic made vaccine over foreign produced. HF: Lack of health belief models with effective health promotion strategies resulted in vaccine hesitancy.
	2020	Wang et al. [73]	2058	Adults	China	91.30%	AF: Being male, married, perceiving a high risk of infection, valuing a doctor’s recommendation, believing in the efficacy of the vaccine or being vaccinated for influenza in the past season. HF: Female gender, lack of influenza vaccination in the past, less perceived risks of COVID-19 and being married resulted in vaccine hesitancy.
	2020	Zhang et al. [74]	1052	Children below 18 years of age	China	72.60%	AF: Support from a family member, perceived behavioral control related to positive attitude from parents towards vaccinating their children. HF: Higher exposure to negative content regarding the vaccine was associated with parental rejection of the vaccine for their children.
	2020	Wang et al. [75]	806	Adult nurses	Hong Kong	40%	HF: Lack of trust in government institutions and less intention to accept influenza vaccination in the past resulted in COVID-19 vaccine hesitancy.
	2020	Harapan et al. [76]	1359	Adults	Indonesia	93.30%	AF: Exposure to COVID-19 information, being a HCW and increased perceived risk of infection resulted in COVID-19 vaccine acceptance. HF: Lower perceived risks about COVID-19 among retired/older individuals and lack of knowledge about the benefits of the vaccine resulted in vaccine hesitancy.
	2020	Al Mohaitheif and Badhi [77]	992	N/A	Saudi Arabia	64.70%	AF: Older individuals, individuals who are married, having high educational levels, and employed in government sector resulted in vaccine acceptance. HF: Lack of confidence and perceived risks of vaccine side effects demonstrated vaccine hesitancy.
	2020	Dror et al. [78]	388	Doctors, general population, nurses	Israel	78.1%, 75%, 61.1%	AF: Having a child, acceptance of recent most influenza vaccine, or being in the healthcare profession increased vaccine acceptance. HF: Not caring about the harmful effects of COVID-19 resulted in vaccine hesitancy.
	2020	Wong et al. [79]	1159	Adults (General Population)	Malaysia	94.30%	AF: The willingness to pay for the vaccine was influenced by no affordability barriers as well as by socio-economic factors, such as higher education levels, professional and managerial occupations and higher incomes.
Europe	2021	Fedele et al. [80]	Not reported	Population of parents	Italy	27%	HF: Safety concerns in 76% parents. Females, lower education level and younger age were associated with non-adherence to vaccination.
	2021	Di Gennaro et al. [81]	1723	Healthcare workers	Italy	67%	AF: Perceived benefits about the health belief models and health promotion strategies resulted in vaccine acceptance. HF: Lack of trust in vaccine safety, inadequate information regarding vaccine and misinformation on social media encapsulated as the main social and behavioral contributors for vaccine hesitancy.
	2021	Riccio et al. [82]	7605	Adults (General Population	Italy	81.9%	AF: COVID-19 vaccine acceptance was associated with female gender, trust in institutions and personal beliefs about the benefits of getting vaccination. HF: Unemployed individuals and those with a lack of influenza vaccination history demonstrated vaccine hesitancy.
	2021	Aurilio et al. [83]	531	Adults (Nurses)	Italy	91.5%	AF: Female sex and confidence in vaccine efficacy were related to vaccine acceptance. HF: Poor understanding about the need to vaccinate, lack of confidence in vaccines and low educational levels resulted in vaccine hesitancy.
	2021	Guaraldi et al. [84]	1176	Adults (Type 2 Diabetes Mellitus patients)	Italy	85.8%	AF: Social and behavioral factors such as older age, male gender, high educational development and influenza vaccination history were evaluated to be associated with vaccine acceptance. HF: Having experienced adverse effects from past vaccinations resulted in vaccine hesitancy.
	2021	Guiseppe et al. [85]	481	Adults	Italy	84.1%	AF: Perceived risks of getting COVID-19 were prevalent in females, younger individuals and those who believed that COVID-19 is a severe disease. HF: Being male, being unmarried and less perceived benefits of getting vaccinated resulted in vaccine hesitancy.
	2021	Ikiisik et al. [86]	384	General Population	Turkey	51.6%	AF: Perceived benefits of getting vaccinated and high trust in HCWs demonstrated vaccine acceptance. HF: Perception of vaccine risks and younger age were observed to be the main contributors for vaccine hesitancy.
	2021	Yigit et al. [87]	343	Healthcare Workers	Turkey	50%	AF: Men demonstrated high vaccine acceptance. Individuals who were employed and older people exhibited vaccine acceptance. HF: The younger the age, the higher the vaccine hesitancy was reported.
	2021	Yurttas et al. [88]	732 patients with rheumatic diseases, 763 general public and 320 healthcare providers	Patients with rheumatic diseases, general population and healthcare providers.	Turkey	29.2% (patients with rheumatic diseases), 34.6% (general population), 52.5% (healthcare providers)	HF: Unknown scientific results, perceived vaccine side effects and lack of trust in government institutions were major factors for the low vaccine-acceptance rates.
	2021	Williams et al. [89]	3436 (1st survey); 2016 (2nd survey)	Adults (General Population)	Scotland	74%, (1st survey); 78% (2nd survey)	AF: Participants of white ethnicity, and individuals with high income levels and high education levels resulted in vaccine acceptance. HF: Black. Asian and minority ethnic groups with lower income and educational levels demonstrated vaccine hesitancy.
	2021	Fakonti et al. [90]	437	Nurses and Midwives	Cyprus	30%	HF: Fear of side effects, female gender, younger age, lack of history of influenza vaccination and working in private sector resulted in vaccine hesitancy.
	2021	Papagiannis et al. [91]	340	Health Professionals	Greece	78.5%	AF: Less fear of vaccine side effects and adequate information received from Greek public health authorities effected vaccine-acceptance rate. High vaccination coverage and absence of fear over vaccine safety were also responsible for high vaccine acceptance.
	2021	Schwarzinger et al. [92]	1942	Adults (working population)	France	71.2%	HF: Vaccine refusal was associated with low educational level, chronic diseases, female gender, age and lower perceived severity of COVID-19.
	2021	Gagneux-Brunon et al. [93]	2047	Adults (Healthcare workers)	France	76.90%	AF: Older age, male gender, and perceived fear about COVID-19 increased vaccine-acceptance rates. HF: Hesitancy to the vaccine relating with female gender, being a nurse or suffering from a chronic medical condition.
	2021	Sherman et al. [94]	1500	Adults (General Population)	UK	64.00%	AF: Positive beliefs and attitudes for the COVID-19 vaccine were associated with vaccine acceptance. HF: General lack of knowledge in the vaccine and belief in side effects was related to vaccine hesitancy.
	2020	Neumann-Bohme et al. [95]	1000	Adults	Denmark, UK, Portugal, Netherland, Germany, France, Italy	80%, 79%, 75%, 73%, 70%, 62%, 77.30%	AF: Men above 55 years with high perceived risks about getting COVID-19 and benefits of vaccination resulted in vaccine acceptance. HF: Vaccine hesitancy dependent on female gender, younger age, and a lack of trust in a vaccine prepared very quickly.
	2020	Freeman et al. [96]	5114	Adults (General population)	UK	71.70%	AF: Age, gender, ethnicity income and region matched with vaccine acceptance. HF: Vaccine hesitancy associated with negative beliefs including mistrust, conspiracy theories and negative support of doctors.
	2020	Bell et al. [97]	1252	Adults (General Population)	UK	89.10%	AF: Protection of own self and family members, high trust in vaccines, scientific literature and HCWs, to stay safe to look after children and the need for stopping social distancing resulted in vaccine acceptance. HF: Race, ethnicity and low-income households most prominently related to vaccine hesitancy. Also relating to vaccine rejection was mistrust in a rapidly developed vaccine.
	2020	Salali and Uysal [98]	1088; 3936	Adults	UK, Turkey	83%; 77%	AF: Willingness of participants to get vaccinated against the virus and high levels of education helped enhance vaccine acceptance. HF: Less public health campaigns demonstrated vaccine hesitancy.
	2020	Detoc et al. [99]	3259	Adults	France	77.60%	AF: Older age, male gender, perceived risks about getting infected with the coronavirus and being a HCW increased vaccine acceptance. HF: Younger age, female gender, anxiety and misconceptions about COVID-19 and associated risk factors with the vaccine resulted in vaccine hesitancy.
	2020	Ward et al. [100]	5018	Adults (General Population)	France	76%	AF: Older individuals, men and individuals with high educational levels accepted the vaccine. HF: Believing that a vaccine produced quickly would be unsafe resulted in vaccine hesitancy.
	2020	La Vecchia et al. [101]	1055	Aged 15–85 years (General Population)	Italy	53.70%	AF: Older age, occupation and willingness to be vaccinated against influenza were related to the intention to be vaccinated against COVID-19. HF: Vaccine mistrust, less qualified individuals and those with lower educational levels demonstrated vaccine hesitancy.
	2020	Barello et al. [102]	735	Adults (University Students)	Italy	86.10%	AF: High levels of trust in health promotion strategies and government institutions increased vaccine acceptance. Students having high levels of education demonstrated high vaccine acceptance. HF: Fears of vaccine safety and side effects resulted in vaccine hesitancy.
North America	2021	Waters et al. [103]	342	Adolescents and young adults (15–39 years)	United States	63%	AF: Male gender and those having high educational backgrounds resulted in vaccine acceptance. HF: Female gender and individuals with a high school education or less, reported high vaccine hesitancy.
	2021	Mascarenhas et al. [104]	248	Dental students	United States	56%	HF: Lack of trust in public health experts, perceived risks of vaccine side effects were major contributors affecting the vaccine-acceptance rate.
	2021	Viswanath et al. [105]	1012	Adults	United States	65%	HF: Vaccine hesitancy was based on risks associated with the COVID-19 vaccine, exposure to social-media platforms and ethnicity along with less education levels.
	2020	Pogue et al. [106]	316	General Population	United States	68%	HF: Efficacy, length of testing and perceived vaccine side effects lead towards vaccine hesitancy.
	2020	Fisher et al. [107]	1003	Adults	United States	56.90%	HF: Younger age, black race, low education attainment and lack of information resulted in vaccine hesitancy.
	2020	Malik et al. [108]	672	Adults (General Population)	United States	67.00%	AF: Males, older adults, Asians, individuals with high educational levels were more willing to accept the vaccine. HF: Females, young adults, racial ethnic groups and those having less education demonstrated vaccine hesitancy.
	2020	Reiter et al. [109]	2006	Adults (General Population)	United States	68.50%	AF: Willingness to be vaccinated was related to healthcare provider’s advice, political understanding, and knowledge about vaccine harms.
	2020	Taylor et al. [110]	1902; 1772	Adults (General Population)	Canada, United States	80.0%; 75.0%	HF: Vaccine rejection was strongly influenced by mistrust of vaccine benefits and by worries about unforeseen future effects, concerns about commercial profiteering from pharmaceutical companies, and preferences for natural immunity.
Australia	2021	Seale et al. [111]	1420	Adults (18 years and above)	Australia	80%	AF: Females, individuals aged 70 years and above, individuals with private health insurance and those suffering from chronic diseases demonstrated vaccine acceptance. Family support greatly increased vaccine acceptance. HF: Males and individuals having age between 18 to 29 years demonstrated vaccine hesitancy.
	2021	Rhodes et al. [112]	2018	Adults (Parents and Guardians)	Australia	75.80%	AF: Women, men and generally people with higher socioeconomic status were related for vaccine acceptance. HF: Vaccine hesitancy was associated with a younger age, educational level and knowledge about the COVID-19 vaccine.
Africa	2021	Adeniyi et al. [113]	1308	Adults (healthcare workers)	South Africa	90.1%	AF: Social factors including high levels of education were associated with vaccine-acceptance rates.
	2021	Saeid et al. [114]	2133	Medical Students	Egypt	90.5%	AF: Female students, students in medicine and physiotherapy and students who had high income and socioeconomic status demonstrated high vaccine acceptance. HF: Inadequate information relating to vaccine effects and insufficient information of the vaccine itself led to vaccine hesitancy.
	2020	Nzaji et al. [115]	613	Adults (healthcare workers)	Democratic Republic of Congo	27.70%	AF: Male HCWs, particularly doctors and having a positive attitude towards COVID-19 vaccine resulted in vaccine acceptance. HF: Misinformation about vaccine safety and side effects on social networks were responsible for vaccine hesitancy.
South America	2021	Cerda and Gracia [116]	370	General population	Chile	49%	HF: Perceived side effects including immunity and less awareness by the government authorities about vaccine benefits were evaluated as reasons for vaccine hesitancy.
	2020	Sarasty et al. [117]	1050	Adults (General Population)	Ecuador	97%	AF: Willingness to pay was associated with income, employment status and the probability of hospital charges if the virus was contracted.

## Data Availability

Data is contained within the article.
